# Addressing the unmet needs in patients with type 2 inflammatory diseases: when quality of life can make a difference

**DOI:** 10.3389/falgy.2023.1296894

**Published:** 2023-11-09

**Authors:** Enrique Gómez de la Fuente, Isam Alobid, Iñigo Ojanguren, Virginia Rodríguez-Vázquez, Beatriz Pais, Víctor Reyes, Miriam Espinosa, África Luca de Tena, Irantzu Muerza, Eduard Vidal-Barraquer

**Affiliations:** ^1^ Department of Dermatology, University Hospital Alcorcon Foundation, Madrid, Spain; ^2^ Rhinology and Skull Base Unit, Department of Otorhinolaryngology, Hospital Clínic, IDIBAPS, CPERES, Barcelona University, Barcelona, Spain; ^3^ Pneumology Service, University Hospital Vall d’Hebron, VHIR, CIBERES, Autonomous University of Barcelona, Barcelona, Spain; ^4^ Allergology Service, University Hospital Complex of Santiago, University of Santiago Compostela, A Coruña, Spain; ^5^ Quality and Patient Safety Unit, Quality Subdirectorate, Healthcare Area of Santiago de Compostela y Barbanza, Servizo Galego de Saúde, Santiago de Compostela, Spain; ^6^ Regional Ministry of Health of Andalusia (CSJA), Adviser, Sevilla, Spain; ^7^ Asociación Española de Esofagitis Eosinofílica (AEDESEO), Madrid, Spain; ^8^ Asociación de Afectados por la Dermatitis Atópica (AADA), Madrid, Spain; ^9^ Asociación de Apoyo a Personas Afectadas por el Asma de Bizkaia (ASMABI), Bilbao, Spain; ^10^ Alira Health, Barcelona, Spain

**Keywords:** type 2 inflammation, quality of life, unmet needs, coexisting pathologies, multidisciplinary management

## Abstract

**Background:**

Patients with asthma (AS), atopic dermatitis (AD), allergic rhinitis (AR), eosinophilic esophagitis (EoE), chronic rhinosinusitis with nasal polyps (CRSwNP), chronic urticaria (CU), non-steroidal anti-inflammatory drugs-exacerbated respiratory disease (N-ERD), and certain phenotypes of chronic obstructive pulmonary disease (COPD), among others, have a common underlying pathogenesis known as Type 2 inflammation (T2i). These diseases often coexist with other T2i conditions and have a substantial impact on the quality of life (QoL) of patients. However, limited data on patients’ experiences, perspectives, and current management of T2i diseases have been published thus far.

**Aims:**

This survey, promoted by the patient-driven T2i Network Project, aimed at identifying the common drivers and challenges related to the QoL of patients with T2i diseases by putting the patient's perspective at the force and including it in the design of new care strategies.

**Methodology:**

An anonymous online survey was carried out through convenience sampling between May and June 2023. The survey was codesigned by members of different patient associations, healthcare professionals and healthcare quality experts, and implemented using EUSurvey and distributed through eight patient associations from Spain. The survey consisted of 29 questions related to the participant's sociodemographic features, a series of self-reported multiple choice or rating scale questions, including diagnosis, QoL measures, disease severity, healthcare resource utilization, and quality of care.

**Results:**

The survey included 404 participants, members from eight patient associations, the majority of whom had moderate-to-severe self-reported disease severity (93%) and one or more coexisting pathologies related to T2i (59%). Patients with more than one pathology had a significantly greater impact on QoL than those with only one pathology (*p* < .001). Participants with self-reported severe symptoms reported significantly worse QoL than those with mild-to-moderate severity (*p* < .001). More than half of the patients (56%) felt constantly bothered by the unpredictability of their illness caused by potential exposure to known or unknown disease triggers. The lack of coordination between specialists and primary care was also expressed as an area of dissatisfaction by participants, with 52% indicating a complete lack of coordination and 21% indicating an average coordination.

**Conclusion:**

This article reports the initial findings of a patient-led initiative, which highlights the common QoL challenges faced by individuals with type 2 inflammation-related diseases and emphasizes the importance of further clinical research to improve the management of this patient group. Considering the significant impact on QoL, a multidisciplinary approach integrated into new healthcare protocols has the potential to improve patient management and QoL, shorten the time to diagnosis and reduce healthcare resource utilization.

## Introduction

Certain phenotypes of AS, AD, AR, EoE, CRSwNP, CU, induced or spontaneous, N-ERD and certain phenotypes of COPD, usually share the common pathophysiology of T2i (1–5). The key drivers of a T2i response, which can be both systemic and local, encompasses chronic dysregulation of both innate and adaptive cell types, with the activation of the T-helper 2 (TH2) pathway and its downstream production of Type 2 interleukins (ILs) such as IL-4, IL-5, and IL-13. These cytokines play a pivotal role in the pathobiology of T2i driving immune dysregulation, triggering eosinophilic-mediated inflammation, epithelial barrier dysfunction, and mucus production among others (2, 6, 7). Ultimately, the persistence of T2i creates a vicious cycle with the escalation of the inflammatory response and exacerbating clinical symptoms (8).

The prevalence of T2i diseases has increased globally. Asthma, AR, AD, and CRSwNP currently affect up to 20% of the general population (9, 10). Moreover, a high proportion of patients (approximately 50% to 70%) with moderate-to-severe AS, CRSwNP, or AD were found to co-exist with at least one T2i disease, and up to 36% with two T2i diseases (11).

Moreover, studies have reported that patients with moderate-to-severe T2i diseases tend to suffer more than patients with mild conditions on most QoL dimensions and are associated with increased healthcare-related direct and indirect costs (9, 12–18). Furthermore, patients suffering from chronic T2i diseases, such as CRSwNP, AS, or AD, report similar unmet needs that could be addressed by the healthcare community to improve clinical and QoL outcomes. For example, the adoption of a multidisciplinary approach in their care management, improving physician coordination, or increasing the awareness of the disease burden (10, 12). However, even if the multimorbidity and interconnectivity of these conditions and the burden on these patients is acknowledged, they are often managed as single entities with little coordination among healthcare professionals (10).

Currently, no holistic tools to assess the QoL burden of patients with T2i diseases are available. Only disease-specific QoL instruments that are tailored to unveil specific challenges and dimensions of some pathologies are available. For instance, AS-specific QoL measurement tools, such as the Asthma Quality of Life Questionnaire-Juniper (AQLQ-J), incorporate exposure to environmental stimuli as a distinctive factor impacting a patient's QoL (13). For CRSwNP, no specific QoL measurement tool exists (14), but the loss of smell and/or taste is a significant QoL factor present in the Sinonasal Outcome Test 22 (SNOT-22) Questionnaire, used to guide healthcare professionals in the clinical management of CRSwNP (15). For adults with AD, one of the specific QoL instruments is the Quality of Life Index for Atopic Dermatitis (QoLIAD), which includes questions related to their personal relationships, such as assessing the shame of these patients when displaying their skin, limitations in clothing choices, and apprehension towards physical contact with others (16). However, the most frequently used in AD is the generic Dermatology Quality of Life Index (DLQI) which also includes dimensions regarding personal relationships, leisure, or impact of treatment (17). Chronic urticaria is assessed using the Chronic Urticaria Quality of Life Questionnaire (CU-Q_2_OL), which also measures personal relationships, including the “looks” dimension and assesses how patients feel about not wearing certain clothes or cosmetics, going to public places, or being embarrassed by symptoms (18). For CU, it is important to measure the degree of unpredictability of disease exacerbations and distinguish between chronic spontaneous urticaria (CSU), with an unknown trigger, and chronic inducible urticaria (CIndU), with a specific trigger, such as cold, pressure, or heat (19). Moreover, in the case of CIndU, patients may experience anaphylaxis shocks, which can have a significant impact on QoL (20). For EoE, the fear of choking when eating and the resulting impact on daily food management are the differentiating QoL factors (21). For COPD, physical limitations are an important feature to be measured and implemented in the McGill COPD QoL questionnaire that includes the assessment of the fatigue dimension (22). For N-ERD, no specific QoL tool is available but patients with this condition are greatly affected by nasal blockage and loss of smell and taste, as well as those with CRSwNP (23). All in all, disease-specific QoL assessments highlight common QoL challenges faced by patients with diseases related to T2i diseases, such as unpredictability and impact on social relationships. The common pathobiology mechanism of such diseases and the fact that those often coexist, calls for a holistic view on the assessment of their QoL.

Limited data on patients’ experiences, perspectives, and current management of T2i diseases have been published. In this context, a group of eight patient associations in Spain has joined efforts and established the T2i Network to contribute to the identification of common drivers and difficulties in the QoL among patients with T2i, including patients’ voices and help with the design of new care strategies for this group of patients. This paper represents the first learnings of the activities performed by this network in order to understand and quantify the common QoL challenges of patients suffering from T2i-related diseases.

## Materials and methods

### Project design and procedures

This project was carried out using an anonymous online survey through convenience sampling between May and June 2023. The survey was implemented using EUSurvey, an online survey-management system built for the creation and publishing of globally accessible forms, supported by the European Commission's Digital Europe Programme (DEP), an interoperability program that guarantees full anonymization. Participation was voluntary and anonymous, and participants were informed about the objectives of the project and provided consent to participate. The survey was distributed through eight Spanish patient associations: the Association of Atopic Dermatitis (AADA), the Association of Chronic Urticaria (AAUC), the Association of Chronic Respiratory Patients (*A tot pulmó*), Spanish Association of Eosinophilic Esophagitis (AEDESEO), the Spanish Association of People with Allergies to Food and Latex (AEPNAA), Spanish Association of Nasal Polyposis (AEPONA), Spanish Association of COPD Patients and Caregivers (EPOC España) and the Spanish Federation of Associations of Allergic Patients and Respiratory Diseases (FENAER). The survey was distributed to all members of patient associations through internal channels (i.e., email) to ensure that a representative sample of patients answered the survey. All data used for this project was collected from the self-reported survey, the clinical history of patients was not accessed nor consulted.

### Survey and data collection

The survey was built by first conducting a patient advisory board with eight representatives of the eight patient associations to understand disease experiences, the care pathway and the QoL of patients with T2i conditions. The insights collected during the patient advisory board were used to design the survey, and the structure and content of the survey were discussed, reviewed, and approved by the above-mentioned Spanish patient associations and an expert team of four physicians, including a dermatologist, a pneumologist, an allergologist, an otorhinolaryngologist and two experts in healthcare quality management from different Spanish hospitals. The survey was developed in Spanish; the English translation is available in the Supplementary Annex.

The survey consisted of a total of 29 questions related to a participant's sociodemographic features, a series of self-reported multiple choice or rating scale questions, including diagnosis, QoL measures, disease severity, questions related to healthcare resource utilization (e.g., yearly medical appointments, hospitalizations, and emergency department visits, number of specialists encountered) and a series of questions related to the quality of care (e.g., time to diagnosis, satisfaction with the information about the disease and about solutions to improve QoL provided by healthcare professionals, number of specialists visited for a condition, level of coordination between specialists and primary care HCPs).

The self-reported diagnoses were collected from a multiple-choice question, including diseases with T2i phenotypes such as, AS, AR, CRSwNP, AD, EoE, COPD, CU (induced or spontaneous), and N-ERD. The survey integrated a question specifically aimed at identifying the presence of allergies, including food, medicine, or environmental. However, it was determined to exercise caution and exclude this information from the study due to concerns regarding potential patient confusion, specifically in distinguishing between intolerance and allergy, as well as the broad scope of the question, which encompassed non-Type two allergies.

The self-reported QoL data were collected from 5-point Likert scale questions (where 5 indicates the highest impact and 1 the lowest) from the survey that resulted in 11 main variables analyzed: depression, anxiety, isolation, sexual-affective relationships, sleep quality, unpredictability, physical limitations, planning difficulties, work absenteeism, treatment efficacy satisfaction, treatment secondary effects.

The self-reported disease severity was collected from a 3-point scale question including the options: mild, moderate, and severe. If the participant had multiple conditions, only the highest severity of the multiple conditions was requested to be reported.

The healthcare resource utilization data were collected from 4-point scale questions (including none, between 1 and 3 times, between 3 and 5 times, and more than 5 times) for yearly hospitalizations and visits to the emergency department and a 6-point scale question (including none, once a year, once every 6 months, once every 3 months, once a month and more than once a month) for yearly medical appointments.

The quality-of-care data were collected with three variables, including time to diagnosis, quality of the information provided by healthcare professionals about the disease and QoL, and the quality of a multidisciplinary care approach. The time to diagnosis was aimed at collecting the time lapse from the first symptom presentation until the disease diagnosis. A 5-point scale question (including less than 2 months, between 2 and 6 months, between 6 months and 2 years, and more than 5 years) was used, and if the participant had multiple conditions, only the longest time to diagnosis from the different conditions was requested to be reported. The satisfaction with the information about the disease and about solutions to improve the QoL provided by healthcare professionals was collected from two questions and measured as dichotomous variables (e.g., Yes/No). The number of specialists visited for a condition was collected from a multiple-choice question, including all relevant specialists. The patient's opinion on the level of coordination between specialists and primary care was collected from a 4-point scale question, including none, average, good, and very good.

### Inclusion criteria

All the analyses were performed with participants that were aged more than 18 years old, resident of Spain, and reported to have at least one T2i disease among AS, AD, AR, EoE, CRSwNP, CU (induced or spontaneous), N-ERD and COPD. As there is not a clear consensus on the number and definition of COPD subtypes (24), to overcome the risk of misidentification of T2i, only patients with COPD and a coexisting T2i disease have been included.

### Statistical analysis

All descriptive [i.e., mean, standard deviation (SD)] and inferential statistical analyses were conducted using RStudio (R v.4.1.0).

A per-patient QoL composite score, the Total QoL (TQoL) was generated by calculating the average across QoL variables measured with a 5-point Likert scale. Before calculating the average, anxiety, depression, isolation, and sexual-affective relationships were grouped into a single variable, the psychological impact generated from the mean of the included variables. Similarly, treatment efficacy satisfaction and treatment secondary effects were also grouped into a single variable, the treatment burden generated from the mean of the two variables.

The Kruskal–Wallis non-parametric test and the *post-hoc* Dunn test for multiple comparisons of severity groups were applied to analyze Likert scale data. Parametric tests were used to perform any analysis on the TQoL. The Analysis of Variance (ANOVA) test was applied to test if there were TQoL differences between self-perceived severity groups and a *post-hoc* analysis using a pairwise *t*-test to identify the groups with significantly different TQoL. A two-way ANOVA test and the means comparison by Tukey's test were applied to analyze the effect of having one T2i disease or having co-existing diseases and the severity of the disease on the TQoL and to test its interaction. In all cases, significance was assessed at an α level of *p *< 0.05, and applied an adjustment for multiple testing (Bonferroni correction) if convenient.

### Ethical considerations

The study was approved by the Local Ethics Committee of a Spanish regional hospital (HUFA/23-79). All participants were informed about the study objectives when completing the survey. The ethics committee approved the exemption of including informed consent, considering the complete anonymization of the survey and the inability to identify respondents in any manner.

## Results

### Patient characteristics

Out of the 404 surveys collected, 43 were excluded for being from patients reporting only a COPD diagnosis and 16 for being from patients reporting only allergies, leaving a total of 345 answers for the analysis.

The mean age of participants was 41.6 (SD = 13.9), of which 68% were female (Table 1). Asthma was the most reported T2i disease among all participants (*n* = 180, 52%), followed by AD (*n* = 156, 45%) and AR (*n* = 125, 36%). Most patients in the study (59%, *n* = 202) reported being diagnosed with more than one T2i disease. The self-perceived severity of the disease among participants was mild for 7%, moderate for 40%, and severe for 53%.

**Table 1:** Overview of patient characteristics.

Patient characteristics				
Total sample	*N* = 345			
Median age (range)	42 (18–97)			
Sex				
Female	68% (*n* = 236)			
Male	31% (*n* = 108)			
Not answered	0% (*n* = 1)			
Coexisting T2ID	Only 1 T2ID		More than 1 T2ID	
All participants (*n* = 345)	41% (*n* = 143)		59% (*n* = 202)	
Asthma (*n* = 180)	15% (*n* = 27)		85% (*n* = 153)	
Atopic Dermatitis (*n* = 156)	30% (*n* = 46)		70% (*n* = 110)	
Allergic Rhinitis (*n* = 125)	5% (*n* = 6)		95% (*n* = 119)	
Eosinophilic Esophagitis (*n* = 87)	43% (*n* = 37)		57% (*n* = 50)	
CRSwNP (*n* = 58)	14% (*n* = 8)		86% (*n* = 50)	
Spontaneous Chronic Urticaria (*n* = 45)	42% (*n* = 19)		58% (*n* = 26)	
Induced Chronic Urticaria (*n* = 10)	0% (*n* = 0)		100% (*n* = 10)	
Aspirin exacerbated respiratory disease (*n* = 17)	0% (*n* = 0)		100% (*n* = 17)	
COPD (*n* = 16)	0% (*n* = 0)		100% (*n* = 16)	
Self-perceived Severity	Mild	Moderate		Severe
	7% (*n* = 25)	40% (*n* = 138)		53% (*n* = 182)

### Quality of life impact of type 2 inflammation diseases

**Figure 1:**
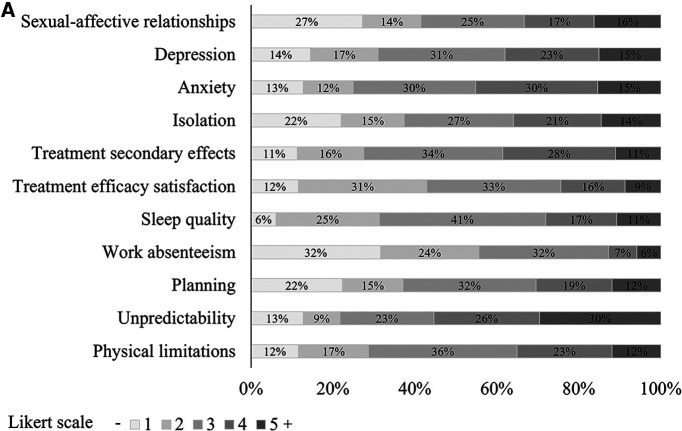

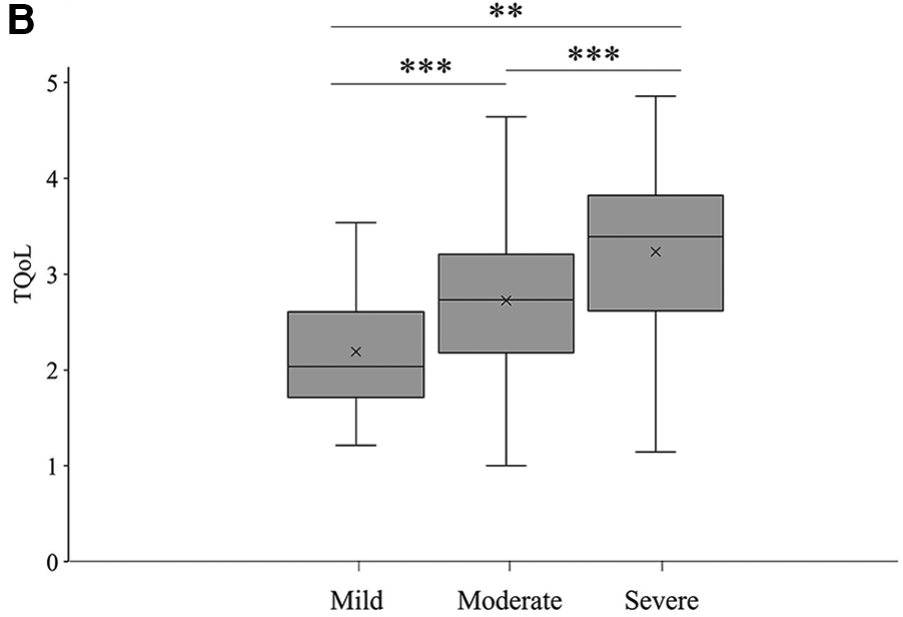

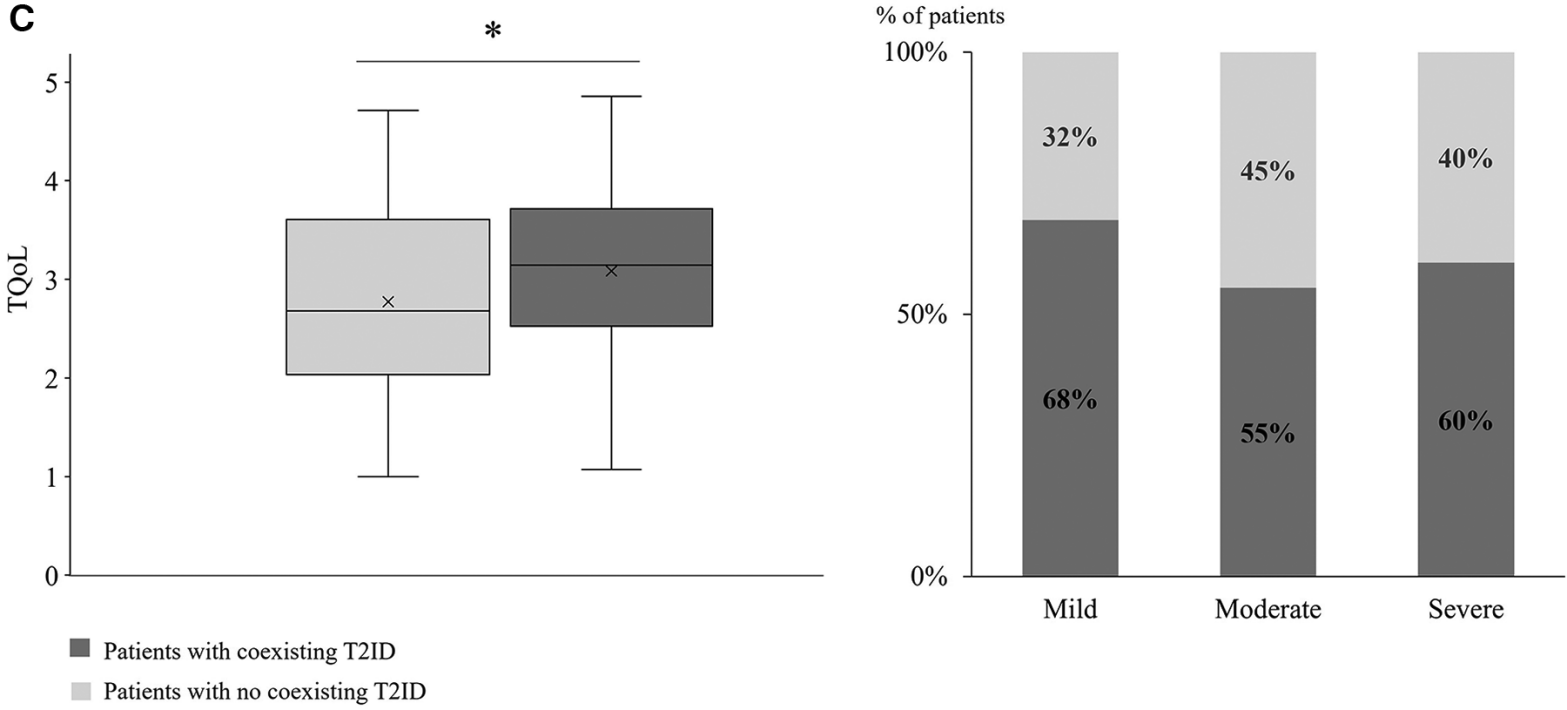
(**A**) Analysis of the QoL impact per dimension. (**B**) T2ID severity impact on QoL (*: *p* < 0.05; **: *p* < 0.01; ***: *p* < 0.001). (**C**) Coexisting T2ID impact on QoL (*: *p* < 0.05; **: *p* < 0.01; ***: *p* < 0.001).

Between 44% and 78% of participants with T2i diseases reported an impact (score ≥3) on each of the QoL dimensions defined in the project, indicating that QoL is commonly and significantly affected among patients with T2i (Figure 1A). The QoL dimension heavily affected in participants was unpredictability, where 56% of participants reported scores of >4, indicating that participants are often or always in a state of alertness to disease triggers. Anxiety and depression were also found to be QoL dimensions affected in patients with T2i diseases (45% and 38% of participants reported scores ≥4, respectively), highlighting the strong psychological burden on these patients. In addition, 39% of participants reported that secondary effects of medications to treat T2i diseases were interfering often or very often with their daily activities of living, indicating low levels of patient satisfaction with the current treatments.

The analysis of the composite TQoL score (Figure 1B), grouping all QoL dimensions (see Methods) by self-reported severity of the disease revealed statistically significant differences among all three groups (*p* < .001), with severe participants presenting the highest impact on their current care with a mean TQoL of 3.2, followed by moderate and mild patients with means of 2.7 and 2.2, respectively (pairwise comparisons: severe vs*.* moderate *p* < .001, severe vs. mild *p* < .001, moderate vs. mild *p* = 0.01). The analysis per single QoL dimension (Supplementary Figure S1) showed significant differences in all QoL dimensions between severity groups except for treatment efficacy satisfaction which is similar between all severity groups.

As shown in Table 1 and Figure 1C, we found that 59% (*n* = 202) of participants in this study had at least two coexisting T2i diseases accounting for 68% (*n* = 17) of mild, 55% (*n* = 76) of moderate and 60% (*n* = 109) of severe patients, suggesting a high prevalence of patients with coexisting T2i diseases. A two-way ANOVA test revealed that the self-reported QoL of patients with coexisting T2i diseases was significantly lower than patients without coexisting T2i diseases (*p* < .001). Moreover, there was not a statistically significant interaction between the severity of the disease (*p* = 0.65) and having one or more coexisting T2i diseases, indicating that patients with coexisting T2i diseases tend to have a decreased QoL independently of the severity of the disease.

**Figure 2:**
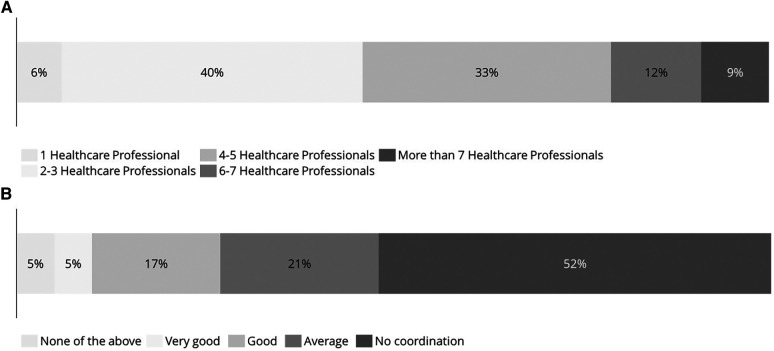
Lack of an effective multidisciplinary approach. (**A**) Number of different healthcare specialists visited since the onset of the disease. (**B**) Perception of coordination between specialists and general practitioners.

According to the survey results, 54% of patients have visited more than three specialists since the onset of their disease and 9% have visited more than seven specialists (Figure 2A). These results indicate that most patients with T2i diseases are managed by multiple specialists throughout their clinical course.

In addition, as observed in Figure 2B, 52% of patients consider that there is a lack of coordination between the specialists and general practitioners and 22% consider that the coordination is good or very good.

Moreover, the survey revealed that patients with T2i diseases have considerable rates of healthcare resource use (Table 2). Eighteen percent of patients have monthly or bi-weekly follow-up visits, 14% of patients have visited the emergency ward more than three times in the last year and 13% have been hospitalized at least once in the last year. These results highlight the need for better coordination among specialists and general practitioners that could potentially reduce healthcare utilization rates, improving healthcare system efficiency and, ultimately, patient QoL.

**Table 2:** Healthcare resource use.

Frequency of healthcare resource use in the last year	
Total sample	*N* = 345
Scheduled visits	
None	7% (*n* = 24)
Once a year	18% (*n* = 61)
Once every 6 months	27% (*n* = 92)
Once every three months	31% (*n* = 107)
Once every month	10% (*n* = 34)
More than once a month	8% (*n* = 27)
Emergency visits	
None	50% (*n* = 174)
Between 1 and 3 times	36% (*n* = 125)
Between 3 and 5 times	6% (*n* = 19)
More than 5 times	8% (*n* = 27)
Hospitalizations	
None	87% (*n* = 301)
Between 1 and 3 times	10% (*n* = 33)
Between 3 and 5 times	1% (*n* = 3)
More than 5 times	2% (*n* = 8)

## Discussion

This article describes the first learnings from a patient-driven initiative led by eight Spanish patient associations (The T2i Network), whose objectives are to understand patients’ perspectives on the burden of T2i diseases, including their QoL impact, and the identification of challenges and potential solutions for better care of these patients. Patient associations have played a central role by leading the project, co-designing the survey, and disseminating it among its members. It is critical that patient associations are involved in such projects as they are committed to raise awareness about the disease, promote research, highlight patients’ unmet needs and are valuable sources of information and education by always being up to date with the latest research on their condition. This article sets a precedent by being the first to promote key stakeholder collaboration between Spanish patient associations, healthcare professionals, and QoL experts to collect quantitative data related to the common QoL challenges faced by patients with T2i.

The initiative was launched by first giving the patient a voice during an advisory board session, where patients with T2i diseases identified their shared challenges related to QoL and care management. The key learnings from the advisory board and the extensive knowledge from medical specialists and QoL experts were the basis to design the survey. As a result, the survey designed for patients with T2i included not only QoL dimensions frequently found in generic QoL tools (e.g., sleep quality, psychological impact, treatment satisfaction, physical limitations, work absenteeism etc.), but QoL dimensions, such as unpredictability and planning as well. Unpredictability and planning were included in the survey as they play a critical role in patients with T2i diseases. These patients are usually exposed to known or unknown triggers that can depend on weather conditions or specific environmental factors, such as allergens or smoke. Moreover, patients are subject to unexpected disease exacerbations or flares, even in patients with well-controlled symptoms, increasing unpredictability (25). Therefore, planning daily activities carefully is a must for these patients and ensuring they are well-educated in managing disease triggers, if those are recognizable.

Even though the majority of the disease phenotypes included in the survey are associated with T2i, it is important to note that the pathogenesis of T2i is not present in all manifestations of the included diseases. In fact, a major unmet need in clinical practice is the lack of consensus on the use of specific biomarkers to identify T2i phenotypes to guide more effective and tailored treatments (26). For this survey, all phenotypes were assumed to be classified as T2i, except for COPD phenotypes that required a coexisting T2i disease to be considered as T2i phenotype. Although this could be a limitation of the study, the majority of AS and CRSwNP cases correspond to T2i phenotypes ranging from 70% in AS (27) to 87% in CRSwNP (28). Additionally, the presence of coexisting pathologies related to T2i, can be used as a proxy to assign a T2i phenotype.

Several studies have explored the QoL and management of patients affected by T2i diseases and their coexistence (8, 10, 29–31). The European Patient Needs and Suggestions on Chronic T2i of Airways and Skin (EUFOREA) white paper, a qualitative study that incorporated 81 interviews of patients with AS, CRSwNP, and/or AD, found that the most prominent shared challenges among patients were the lack of a definitive cure and insufficient coordination among HCPs (10). The EUFOREA study also identified common QoL challenges that were included in our survey design, such as physical limitations (35% reported scores ≥4), unpredictability (56% reported scores ≥4), impaired sleep (28% reported scores ≥4), and psychological impact (38% reported scores ≥4) (10). Similarly, we found that 52% of patients perceive a lack of coordination between HCPs, and that 25% of patients express high levels of dissatisfaction (scores ≥4) with the efficacy of their treatment. Our study not only supports the results of EUFOREA but also provides stronger evidence given the quantitative approach that was followed and a more comprehensive analysis with the inclusion of nearly 400 patients.

The outcomes revealed in this study emphasize the necessity for innovative strategies to tackle the unmet needs and shared challenges faced by patients with T2i diseases. These findings highlight the need for a novel healthcare management approach tailored specifically for these patients. In fact, our results suggest that the more severe the manifestation of the disease, the more likely patients will experience a stronger impact on all dimensions of QoL. Therefore, patients with T2i should be managed with a special focus on their QoL and distinct management approaches should be envisaged depending on the severity of the disease. An additional finding of this study is that the coexistence of two or more T2i pathologies, independently of the severity of the disease, decreases patients’ QoL and consequently, a coordinated multidisciplinary approach for the management of these patients is critical. However, coexisting T2i diseases are often managed independently without a coordinated multidisciplinary approach, resulting in poor disease control, persistent symptoms, and poor QoL (8, 10, 32). The results of this survey also indicate that 35% of patients obtained a final diagnosis of one of their T2i diseases after 2 or more than 5 years (see Supplementary Figure S2) and that 63% of patients visited more than three specialists since the onset of their disease. These results highlight again that there is a need for a trained and coordinated multidisciplinary team of HCPs to improve time to diagnosis of coexisting diseases and optimize treatment management and drug prescription (32). Moreover, the survey showed that physical limitations and the psychological impact are two important dimensions to consider in these patients, indicating that a multidisciplinary team should include psychologists and rehabilitators (10). All in all, our results emphasize the need of a multidisciplinary approach for the management of patients with T2i diseases that would not only improve patient outcomes and their QoL, but also could decrease healthcare resource utilization by reducing the time to diagnosis, optimizing the treatment management plan and reducing the number of visits to specialists.

Several studies have shown that certain phenotypes of food allergies [i.e., immunoglobulin E (IgE)-mediated] are associated with T2i and that their prevalence is high (33, 34). Although food allergy was included in our survey as an option to select among the T2i diseases, we did not include it in our main analysis to avoid a potential bias of misidentification of self-reported T2i among respondents. This potential bias was mainly due to the risk of patient confusion between an immune-mediated allergy and a non-immune mediated food intolerance. Nonetheless, the survey showed that 33% of patients self-reported having food allergies. The impact of IgE-mediated food allergies on QoL has been reported to be significant, mainly due to the stress resulting from daily management and avoidance of allergens (35) which is in line with our results related to unpredictability where 56% of participants are often or always in a state of alertness due to triggers associated with their T2i pathologies.

While there are specific QoL assessment tools available for nearly all described pathologies, these tools do not account for the presence of coexisting diseases. A validated tool to measure QoL in such patients is therefore needed to provide the medical community a guide on how to assess the real impact on the QoL of these patients and to develop novel patient management approaches with a real impact on patients. The present project provides the basis for the development of such a tool, hoping that future research will improve the outcomes and the QoL of patients with T2i diseases.

## Conclusion

This article describes the first learnings from a patient-driven initiative led by The T2i Network, showing that individuals who suffer from diseases related to T2i face similar and significant challenges related to their QoL. The impact of QoL among these patients is influenced by two significant factors. Firstly, the severity of their illness amplifies the impact on QoL. Secondly, the presence of coexisting T2i pathologies further worsens the negative effects on QoL. Further clinical research and development of specific tools to assess the QoL of patients with T2i are needed to better understand the characteristics of this group of patients, improve their current management and cover their unmet needs and challenges. Given the complexity of these patients and their significant impact on QoL, there is a clear need to adopt a multidisciplinary approach for their management. This multidisciplinary approach has the potential to improve patient outcomes and QoL, shorten the time to diagnosis, and considerably reduce healthcare resource utilization.

## Data Availability

The raw data supporting the conclusions of this article will be made available by the authors, without undue reservation.

## Supplementary material

ANNEX I

Survey on quality of life for patients with type 2 inflammatory diseases.
