# Supplementary material for: Addressing the unmet needs in patients with type 2 inflammatory diseases: when quality of life can make a difference
Source: Front Allergy. 2023 Nov 9;4:1296894. doi: 10.3389/falgy.2023.1296894 (PMC10680168; doi:10.3389/falgy.2023.1296894)
Supplement: Supplementary file 1 [file Datasheet1.docx]

**Annex I: Survey on Quality of Life for Patients with Type 2 Inflammatory Diseases**

T2i is an excessive immune response to allergens or other triggering factors. Recent studies indicate that T2i underlies various atopic, allergic, and inflammatory diseases, such as asthma, atopic dermatitis, eosinophilic esophagitis, chronic urticaria, chronic rhinosinusitis, prurigo nodularis, chronic obstructive pulmonary disease, among others.

The objectives of this questionnaire are:

- Identify common barriers and challenges of these type 2 inflammatory diseases.
- Study the impact these diseases have on the quality of life of patients.
- Understand unmet needs in the management and care of T2i.

Your responses are anonymous and confidential. By answering this questionnaire, you agree to participate in this national study investigating the effects of T2i on patients' quality of life and promoting coordinated and integrated healthcare for these conditions. The questionnaire consists of 29 quick-response questions and should take about 10 minutes to complete. Please answer all the questions and click "Submit" when finished.

Thank you for your cooperation.

Before we begin,

Please indicate your gender:

a. Male

b. Female

c. Undetermined

Please indicate your age: (numeric field, two digits)

1. I have been diagnosed with: (You can select more than one option)

a. Asthma

b. Atopic dermatitis

c. Chronic rhinosinusitis with nasal polyps

d. Chronic rhinosinusitis without nasal polyps

e. Recurrent otitis media

f. Chronic spontaneous urticaria

g. Chronic inducible urticaria

h. Chronic obstructive pulmonary disease

i. Eosinophilic esophagitis

j. Prurigo nodularis

k. Food allergies (e.g., peanuts)

l. Environmental allergies (e.g., pollen)

m. Medication allergies (e.g., amoxicillin)

n. Aspirin-exacerbated respiratory disease

o. Allergic rhinitis

1. What was the maximum time from the onset of the first symptoms to the diagnosis of the previously selected disease? (If you have multiple conditions, please consider the one that took the longest to be diagnosed)

a. Less than 2 months

b. Between 2 months and 6 months

c. Between 6 months and 2 years

d. Between 2 and 5 years

e. More than 5 years

1. Please indicate, in your opinion, the severity of the previously selected disease: (If you have multiple diseases, please consider the one you consider the most severe)

a. Severe

b. Moderate

c. Mild

1. Taking into account the previously selected disease or set of diseases, please indicate your level of agreement or disagreement with the following statements:

|  | Strongly Agree | Agree | Neutral | Disagree | Strongly Disagree |
| --- | --- | --- | --- | --- | --- |
| The symptoms of my disease or set of diseases are very intense and limiting |  |  |  |  |  |
| Constantly, the symptoms of my disease or set of diseases worsen, and I have to take extraordinary measures |  |  |  |  |  |
| The symptoms of my disease or set of diseases are persistent and do not cease |  |  |  |  |  |

1. In your opinion, please indicate how good or bad your overall health has been during the past year due to the previously selected diseases

a. Very bad

b. Bad

c. Normal

d. Good

e. Very good

1. Please indicate your level of satisfaction or dissatisfaction with how your current treatment alleviates your symptoms:

a. Very unsatisfactory

b. Unsatisfactory

c. Somewhat satisfactory

d. Satisfactory

e. Very satisfactory

1. To what extent do the side effects of your treatment interfere with your physical health and/or daily activities?

a. Not at all

b. Very little

c. Somewhat

d. A lot

e. Very Much

1. During the past year and due to any of the previously selected diseases, how frequently...

|  | None | Between 1 and 3 times | Between 3 and 5 times | More than 5 times |
| --- | --- | --- | --- | --- |
| …have you had to go to the emergency room? |  |  |  |  |
| ...have you had to be hospitalized? |  |  |  |  |

1. Since the onset of symptoms until today, please select the specialists you have visited due to any of the previously selected diseases. (You can select more than one option)

a. Allergy

b. Primary care

c. Dermatology

d. Endocrinology

e. Clinical immunology

f. Internal medicine

g. Pulmonology

h. Neurology

i. Otorhinolaryngology

j. Psychology

k. Psychiatry

l. Rehabilitation

m. Rheumatology

n. Other

1. During the past year, how frequently have you had scheduled medical visits with a healthcare professional due to any of the previously selected diseases?

a. None

b. Once a year

c. Once every six months

d. Once every three months

e. Once a month

f. More than once a month

1. During the past month, considering any of the previously selected diseases, how would you rate the overall quality of your sleep?

a. Very good

b. Quite good

c. Average

d. Quite poor

e. Very poor

1. During the past month and due to any of the previously selected diseases, how frequently...

|  | Always | Almost Always | Sometimes | Rarely | Never |
| --- | --- | --- | --- | --- | --- |
| …health or emotional problems related to your disease have hindered your daily activities? |  |  |  |  |  |
| …you have had to be on alert for possible triggers of your disease or allergens (e.g., rain, dust...)? |  |  |  |  |  |
| …you have had to change your plans and/or work, social, or family commitments? |  |  |  |  |  |
| …health or emotional problems related to your disease have hindered your social or family activities? |  |  |  |  |  |
| …you felt very nervous or anxious? |  |  |  |  |  |
| …you felt discouraged or depressed? |  |  |  |  |  |
| …your condition has negatively impacted your romantic/sexual relationships? |  |  |  |  |  |

1. During the past year, have you had to miss your daily, work, or academic obligations due to any of the previously selected diseases?

a. Never

b. Only occasionally

c. Sometimes

d. Almost always

e. Always

1. Considering the past year, please indicate the response that best reflects your work and/or academic experience:

a. I have not had any issues.

b. Lack of understanding of my clinical situation by colleagues, supervisors, and/or academic staff.

c. Incompatibility between my clinical situation and my work and/or studies.

d. None of the above.

1. Are you regularly asked during consultations how your disease affects your daily life in order to help you find alternatives to improve your condition? (Such as dietary recommendations, exercises, work conditions, etc.)

a. Yes

b. No

1. Have they clearly explained in any of the consultations you attended what your disease consists of, how it should be treated, and what can be expected from the treatment?

a. Very well

b. Well

c. Average

d. No

1. In your opinion, the level of coordination between your primary care doctor and the specialists is:

a. Very good

b. Good

c. Average

d. There is no coordination

e. None of the above

1. In your opinion, the level of listening and understanding from your primary care doctor towards you is:

a. Very good

b. Good

c. Average

d. There is no listening
