# Supplementary figures and images for: Addressing the unmet needs in patients with type 2 inflammatory diseases: when quality of life can make a difference
Source: Front Allergy. 2023 Nov 9;4:1296894. doi: 10.3389/falgy.2023.1296894 (PMC10680168; doi:10.3389/falgy.2023.1296894)

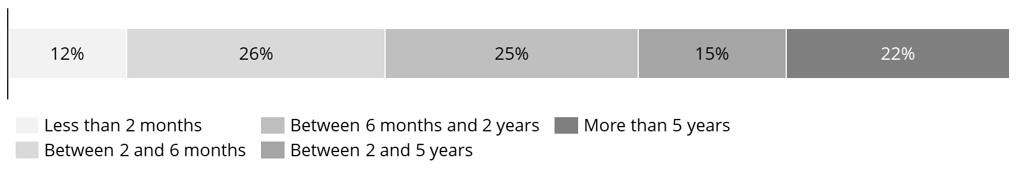

Supplement: Supplementary file 2 [file Image1.jpeg]

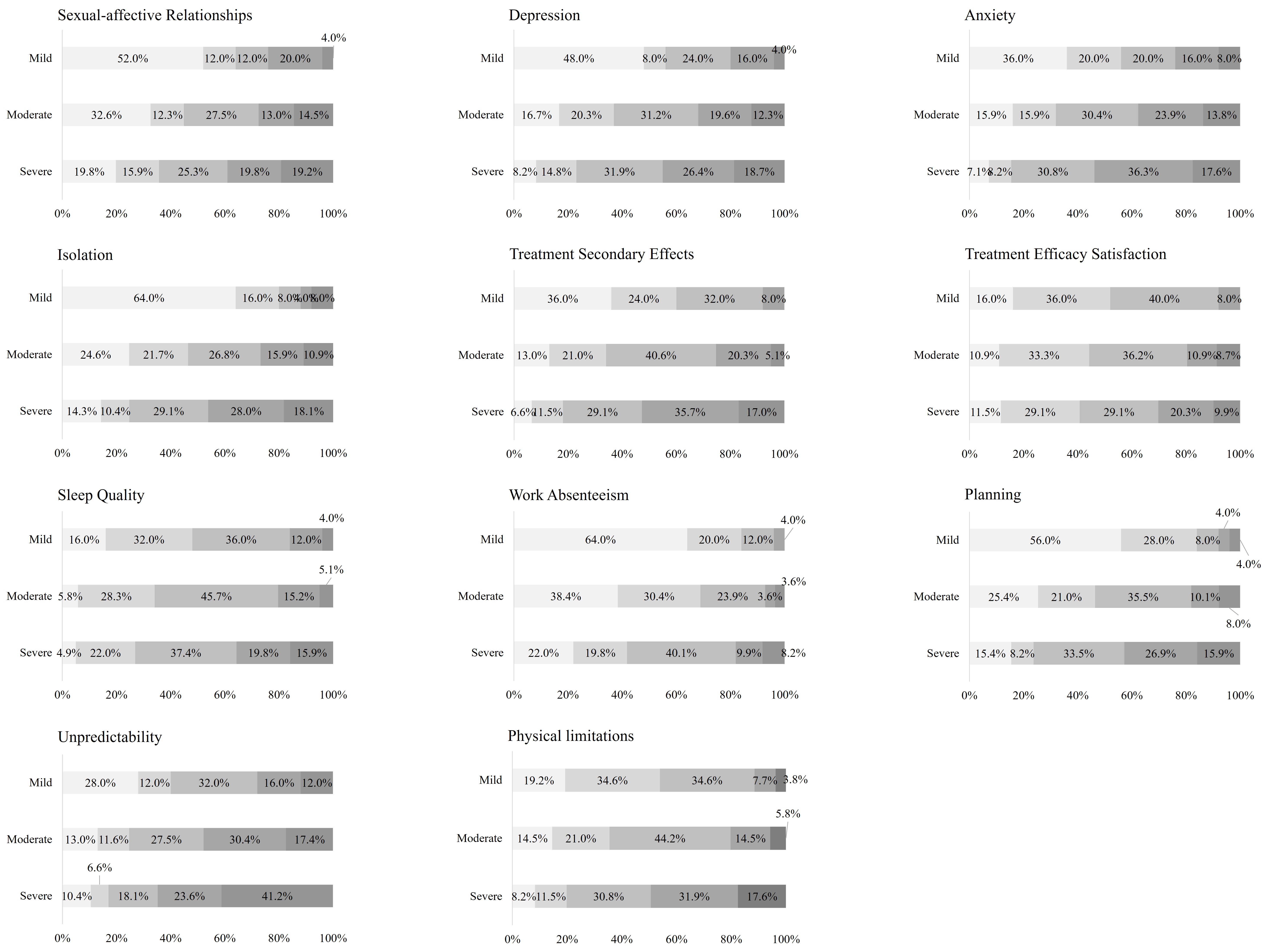

Supplement: Supplementary file 3 [file Image2.jpeg]
